# Participation of women in the health workforce in the fragile and conflict-affected countries: a scoping review

**DOI:** 10.1186/s12960-021-00635-7

**Published:** 2021-08-04

**Authors:** Basnama Ayaz, Maria Athina Martimianakis, Carles Muntaner, Sioban Nelson

**Affiliations:** 1 Lawrence S. Bloomberg Faculty of Nursing, University of Toronto, 155 College Street, Toronto, ON M5T 1P8 Canada; 2 Department of Paediatrics and Scientist, Wilson Centre for research in health professions education, Temerty Faculty of Medicine, University of Toronto, ON 27 King’s College Circle, Toronto, ON M5S 1A1 Canada

**Keywords:** Health workforce, Women, Gender, Conflict-affected countries, Human resources for health

## Abstract

**Introduction and background:**

The full participation of women as healthcare providers is recognized globally as critical to favorable outcomes at all levels, including the healthcare system, to achieving universal health coverage and sustainable development goals (SDGs) by 2030. However, systemic challenges, gender biases, and inequities exist for women in the global healthcare workforce. Fragile and conflict-affected states/countries (FCASs) experience additional pressures that require specific attention to overcome challenges and disparities for sustainable development. FCASs account for 42% of global deaths due to communicable, maternal, perinatal, and nutritional conditions, requiring an appropriate health workforce. Consequently, there is a need to understand the impact of gender on workforce participation, particularly women in FCASs.

**Methods:**

This scoping review examined the extent and nature of existing literature, as well as identified factors affecting women's participation in the health workforce in FCASs. Following Arksey and O'Malley's scoping review methodology framework, a systematic search was conducted of published literature in five health sciences databases and grey literature. Two reviewers independently screened the title and abstract, followed by a full-text review for shortlisted sources against set criteria.

**Results:**

Of 4284, 34 sources were reviewed for full text, including 18 primary studies, five review papers, and 11 grey literature sources. In most FCASs, women predominate in the health workforce, concentrated in nursing and midwifery professions; medicine, and the decision-making and leadership positions, however, are occupied by men. The review identified several constraints for women, related to professional hierarchies, gendered socio-cultural norms, and security conditions. Several sources highlight the post-conflict period as a window of opportunity to break down gender biases and stereotypes, while others highlight drawbacks, including influences by consultants, donors, and non-governmental organizations. Consultants and donors focus narrowly on programs and interventions solely serving women's reproductive health rather than taking a comprehensive approach to gender mainstreaming in planning human resources during the healthcare system’s restructuring.

**Conclusion:**

The review identified multiple challenges and constraints facing efforts to create gender equity in the health workforce of FCASs. However, without equal participation of women in the health workforce, it will be difficult for FCASs to make progress towards achieving the SDG on gender equality.

**Supplementary Information:**

The online version contains supplementary material available at 10.1186/s12960-021-00635-7.

## Introduction and background

Human resources for health (HRH) are central to a quality healthcare system and essential for change and transformation of communities and societies [1–3]. It is estimated that an additional 18 million health workers are required in low- and middle-income countries (LMIC) by 2030 in order to attain the United Nation’s sustainable development goals (SDGs) and the universal health coverage (UHC) [4]. The World Bank (WB) emphasizes gender equality at all levels to transform the distribution of opportunities, resources, and choices for men and women for sustainable development and women’s empowerment in all sectors [5].

The full participation of women as healthcare providers is recognized globally as critical to favorable outcomes at the individual, household and community, and healthcare system level [1, 6]. Simultaneously, the World Health Organization (WHO) acknowledges that systemic challenges, gender biases, and inequities exist for women in the health and social care workforce globally [2] and that fragile and conflict-affected states/countries (FCASs) have additional pressures that require more attention to overcome the existing gender disparities [2, 7, 8]. Moreover, the global strategy on HRH for 2030, adopted by the World Health Assembly, recommends that countries emerging from conflict adopt holistic approaches to improving health outcomes and broader socio-economic development [2].

The average life expectancy in FCASs is 62.2 years compared to 71.4 years globally; 42% of deaths are due to communicable diseases, maternal and perinatal causes, and malnutrition [9]. The maternal mortality rate (MMR) is varyingly high among FCASs. However, targeted interventions in FCASs [9–11] have contributed to an overall reduction in MMR from 424 per 100,000 to 236 between 1990 and 2015 [9]. In addition to interventions targeting maternal and child health, it is vital to control modifiable risk factors for non-communicable diseases, like hypertension, notably twice the global average in FCASs [9]. In some FCASs, including Afghanistan, Iraq, Lebanon, and Mali, the dominance of strong patriarchal structures creates additional health challenges for women by prohibiting women to receive care from male providers [10, 12–16]. In addition, females require permission from male family members to access education and employment in some contexts [7, 12, 15, 17], affecting women’s participation in the health workforce. Thus, women may face difficulty accessing care due to the non-availability of female healthcare providers [13, 14, 17].

**Fig. 1:**
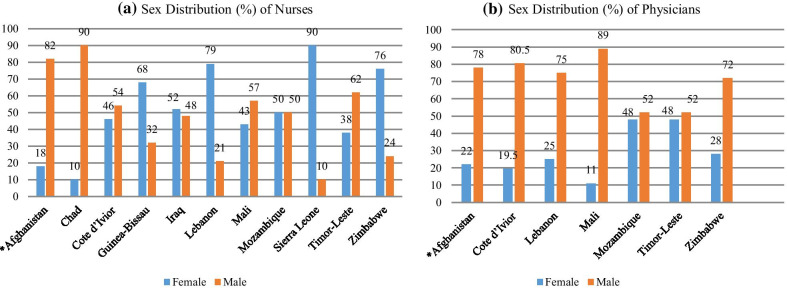
**a** Sex distribution (%) of nurses. **b** Sex distribution (%) of physicians. Global Health Observatory (GHO) on sex distribution, 2020 *Afghanistan’s National Health Strategy 2016–2020. Sex-distributed data of GHO represent different years for different countries. Please see “Additional file 1” for detailed data for different years

Some studies concur on the critical need for a gender-balanced workforce and increased representation of women at all levels, including decision-making levels, in FCASs [1, 2, 7, 14]. In most contexts, women’s employment concentrated in low levels and lower-paid professions such as nursing and midwifery compared to men, who dominate the physician workforce [7]. That said, in some countries, nursing is too dominated by men [18, 19], as is evident in the sex-distributed data from the WHO’s global health observatory (GHO) in Fig. 1a, b [19]. This review seeks to explore the multiple issues affecting gender parity in health workforce participation by summarizing data from various sources from FCASs. It aims to further our understanding of how gender, a key social stratifier, impacts women's participation and career trajectory in the health workforce, particularly in FCASs.

WHO defines gender as “socially constructed roles, behaviors, activities, and attributes that a given society considers appropriate for men and women” [20]. This review focuses on women's participation in the health workforce in FCASs, and considering data presented by GHO, we use the term gender to reference the male and female binary. HRH is used interchangeably with the term health workforce. In order to ensure data consistency across nationally variable terminology and employment categories, we limited our discussion to physicians, nurses, and midwives. These three professions are identified by WHO as professionals based on criteria for education and training, regulation of the professions, and activities and job tasks which are drawn from the International Standard Classification of Occupations and other standards of classifications for social and economic statistics [21]. According to the WB’s harmonized lists for 2018 and 2019, 36 countries or territories [22] are categorized as FCASs based on their financial and security status, meeting the harmonized Country Policy and Institutional Assessment rating of 3.2 or less, and/or the presence of a UN and/or regional peace-keeping or political/peace-building mission during the last three years [9, 22]. Please see “Additional file 2” for the list of these FCASs.

### Research questions

How do women participate and progress to leadership and decision-making positions in the health workforce in FCASs? What enabling and constraining factors shaped the patterns, roles, and outcomes concerning women's employment, retention, and career advancement?

## Methods and analysis

### Design

A scoping review is considered beneficial for examining emerging evidence for the broader question of women's participation in the health workforce in FCASs [23]. We utilized Arksey and O'Malley's five stages framework [24]. The review was reported using Preferred Reporting Items for Systematic reviews and Meta-Analyses extension for Scoping Reviews (PRISMA-ScR) Checklist presented in “Additional file 3” [25]. Following Arksey and O’Malley’s method and the PRISMA-ScR checklist we did not critically appraise the identified studies. We utilized the gender analysis framework developed by Morgan et al. [26] to guide gender/sex integration in the health system’s research, including content, process, and outcomes. This review focuses on assessing the content of the identified studies. The content includes the sex-disaggregated data, utilization of the gender analysis framework, and focused questions based on the WHO's six building blocks of the health system. We present findings from the identified studies for influence of gender on ‘human resources’, which is one of the building blocks [26].

### Identification of sources

A comprehensive search strategy was developed in consultation and assistance from the health sciences librarian at the University of Toronto in 2019 and was re-run in 2020 to maximize sources for review before we publish it [27]. The search focused on the systematic search of published literature in the health sciences databases: Ovid MEDLINE, CINAHL Plus, EMBASE, Scopus, and Web of Science. The search utilized advanced search engines and terms related to women, health workforce, and fragile and conflict-affected states or the name of countries from the WB’s harmonized lists for 2018 and 2019 (“Additional file 4” presents a detailed search strategy for two databases). We also searched for grey literature in Google Advance and other relevant websites of international development agencies, including the WHO, WB, and the Research in Gender and Ethics (RinGs) [28], and ReBUILD consortiums [29]. Scholars working with RinGs and ReBUILD consortiums were consulted to find relevant sources. Hand searches were also conducted of reference lists and websites from identified literature using a snowball search approach.

### Selection of sources

**Fig. 2:**
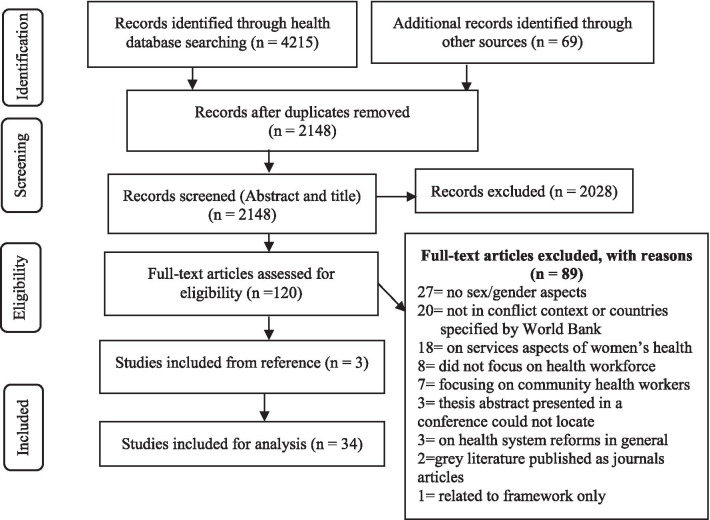
PRISMA flow diagram for screening and selection

Utilizing the Bramer method, systematic de-duplication [30] was conducted in EndNote. A final unique set of records were imported into Rayyan, a web-based software program [31] that streamlined screening, study selection, and data extraction for this review. Two independent reviewers screened the title and abstract, followed by the full-text review of selected sources against the inclusion criteria presented in Table 1. Discrepancies were resolved through team discussion and consensus. The search strategy and selection process results are presented using the PRISMA flow diagram (Fig. 2 presented in the results section). Findings were discussed at regular meetings with all research team members.

**Table 1:** Selection criteria

Inclusion criteria	Exclusion criteria
Research focusing on the processes of recruitment, retention, or leadership in the health workforce in the post-conflict period in health system’s reconstruction in FCASs Provides sex/gender-segregated data in the FCASs All studies, utilizing quantitative, qualitative, and mixed methods and literature review papers Documents from the identified countries (HRH profile) and the international development agencies’ HRH plans and strategies for women’s participation Published after the ending of armed conflicts in each country and with an upper date limit to December 31, 2020 Sources published in English language only	Any study that: is not in the context of FCASs listed by WB harmonized list s2018 and 2019 does not focus on sex/gender aspects does not contain at least one component of recruitment, retention, and promotion or leadership for women in the health workforce

### Data extraction

Data were extracted to a Microsoft Excel sheet from all the selected sources. The information regarding author/s and year, title, source and country, objective/purpose, study design, sample size, participants gender/sex-segregated, framework utilized, key finding, and research gaps indicated by the author/s were analyzed.

## Results

### Description of the identified sources

Of 4284 sources, 34 (23 empirical and 11 grey) were included in full-text review from 11 FCASs (Fig. 2). Some of the identified studies were conducted in multiple countries; we describe results of the countries which are tilted FCASs in the WB’s harmonized lists for the years 2018, and 2019. Tables 2 and 3 detail the 18 primary studies and five review papers, respectively. The 11 grey literature sources included two research project reports from ReBUILD that focussed on the deployment of HRH in Zimbabwe [32] and establishing a responsive and equitable health workforce in FCASs [33]. Two reports from RinGs consortiums to understand gender roles and relations and their effects on health workers' training opportunities and career progression in Zimbabwe [34, 35]. Three documents are from WHO, including a guide to health workforce development in the post-conflict environment [36], a synthesis paper to inform the development of HRH strategy, had a specific section for FCASs [37], and the subsequent global strategy on HRH development 2030 [2]. Two documents from Afghanistan, including the national health strategy 2016–2020, provide a gender-segregated workforce profile and gender equality as the guiding principle for future directions [18] and a Master’s thesis on HRH reconstruction in Afghanistan [38]. One document from the Ministry of Health and Sanitation of Sierra Leone on HRH is included, which provides a comprehensive overview of the HRH situation and its challenges in Sierra Leone [39].

**Table 2:** Details of primary research studies

S. #	Author/s, year	Purpose	Design	Sample/participants	Sex %		Data collection	Frameworks	Country/ies
					M	F			
1.	Dhatt et al. 2017 [1]	Examine the realities, challenges, and opportunities of women's leadership in global health	Mixed methods	64 Health workers	39	61	In-depth interviews and data from international organizations	Thematic analysis	Zimbabwe
2.	Witter et al. 2017 [7]	Understand gender influences on the health workforce in four fragile and post-conflict contexts	Mixed methods	965 Physicians, medical assistance, nurses, midwives and others	44	56	Survey, document review, in-depth interviews and key informant interviews	Gender analysis framework	Sierra Leone and Zimbabwe
3.	Ag Ahmed et al. 2020 [12]	Identify and understand the factors related to shortage and poor retention of skilled health workers in rural health districts of Kayes, Mali	Qualitative	46 5 Physicians 35 Nurses 6 Decision-makers	59	41	In-depth interviews	Thematic analysis	Mali
4.	Alameddin et al. 2016 [13]	Soliciting and synthesizing the voice of PHC and community stakeholders on the HRH recruitment and retention strategies	Qualitative	22 Policy and decision-makers	36	64	Key informant interviews	Thematic analysis	Lebanon
5.	Qarani et al. 2018 [16]	Determined challenges faced by the nursing administration at 17 public hospitals in Kabul	Cross-sectional	86 Nurse mangers and head nurses	79	21	Survey	None	Afghanistan
6.	Witter. S et al. 2017 [44]	Insights from staff remained in services in FCASs; draw lessons to enhancing staff and health system’s resilience	Qualitative	128 Physicians, nurses, midwives, others	27	73	Life histories and in-depth interviews	Thematic analysis	Zimbabwe and Sierra Leone
7.	Witter et al. 2018 [45]	Examine patterns in expressed motivation to join the profession across different settings and cadres to explain their retention	Qualitative	Health Care Providers 103 (F:77, M:26)	25	75	Life histories	Thematic analysis	Sierra Leone, Zimbabwe
8.	Wurie et al. 2016 [46]	Investigate the importance of different motivation factors in rural areas in Sierra Leone to contribute to better decisions on financial and non-financial incentive packages	Qualitative	23 Physicians, nurses, midwives, and community health officers	48	52	In-depth interviews	Thematic analysis	Sierra Leone
9.	Bertone et al. 2018 [47]	Explore how has HRH recruitment policies changed in Timor-Leste (1999–2018), the drivers of change, and their contribution to rebuilding an appropriate health workforce after conflict	Qualitative	20 HRH policy-makers	85	15	Policy analysis and key informant interviews	Thematic	Timor-Leste
10.	Hou et al. 2016 [48]	Understand the labor market dynamics among health workers, including their preferences and concerns, especially regarding their revenues and rural jobs	Cross-sectional	443 175 Physicians 150 Nurses 118 Midwives	42 69 0	58 31 100	Survey	Descriptive	Timor-Leste
11	Gupta & Alfano, 2011 [49]	Investigated gender differences in health workers’ access to non-pecuniary benefits across countries	Cross-sectional	2630 Nurses and midwives Physician	13 69	87 31	Survey	None	Chad, Côte d’Ivoire, Mozambique and Zimbabwe
12.	Russo et al. 2015 [50]	Analyzed the proportion and characteristics of female physicians and implications of the medical workforce’s feminization	Secondary data	331 Physicians	54	46	Primary survey data, secondary analysis	None	Guinea-Bissau, Mozambique
13.	Mashange et al. 2019 [51]	Examine the implementation of deployment policies in Zimbabwe before, during and after the crisis in order to analyze the actual practices used by managers to cope with the crisis	Respective qualitative	95 17 KI 11Managers 67 HWs	59 64 37	41 36 63	In-depth interview Life histories Document analysis	Thematic analysis	Zimbabwe
14.	Jaeger et al. 2018 [52]	Identify challenges and opportunities -in daily work, including factors that influence motivation and social well-being of the healthcare workers	Qualitative	8 Nurses	62	38	In-depth interviews and observations	Thematic analysis	Chad
15.	Squires et al. 2006 [53]	Determine nurses’ priorities for health system reconstruction and the development of the nursing profession	Cross-sectional	744 Nurses	48	52	Survey	Thematic analysis for qualitative comments	Iraq
16.	Squires et al. 2010 [54]	Determine the priorities for health system reconstruction among Iraqi physicians	Cross-sectional	1001 Physicians	71	29	Survey	None	Iraq
17.	Attieh et al. 2018 [55]	Examined residents' and program directors' opinions on motherhood during the residency program	Cross-sectional	98 Residents 22 Program directors	0 82	100 18	Survey	None	Lebanon
18.	Alameddine et al. 2020 [56]	Gain insight into the reasons behind the emigration of Lebanese nurses and understand whether emigration is temporary versus permanent and/or reversible versus irreversible	Cross-sectional	136 Nurses	43	57	Survey	Descriptive	Lebanon

Some studies were conducted in multiple countries; only countries indicated in this table are from the list of WB's harmonized list for 2018 and 2019

**Table 3:** Details of literature review papers

S. no.	Author/s, year	Type of review, objective	Framework	Countries
1.	Roome et al. 2014 [8]	Narrative review Presents a global review of published research on HRM in post-conflict health systems in the past decade (2003–2013)	Analytical framework focusing three functional areas of HRM: workforce supply, workforce distribution, and workforce performance	Post-conflict health systems in general
2.	Percival et al. 2018 [11]	Systematic review Explore if and how health interventions during the post-conflict reconstruction period met gender equity	WHO six building blocks including health service delivery, human resources, health information systems, health system financing, medical products and technologies, and leadership and governance	Mozambique, Timor-Leste, and Sierra Leone
3.	Safi et al. 2018 [17]	Narrative review Provide an overview of interventions used to tackle the critical shortage and distributional imbalances of health workers in rural and remote areas	Thematic Analysis	Afghanistan
4.	Morgan et al. 2018 [40]	Synthesis paper Synthesizes findings from nine studies focusing on four health systems domains, including human resources	WHO building blocks with application of gender and intersectional analysis	LMIC including Zimbabwe
5.	Percival et al. 2014 [43]	Narrative review How gender-sensitive is the health system, and factors need to be considered to build a gender-sensitive health system	WHO six building blocks-health service delivery, human resources, health information systems, health system financing, medical products and technologies, and leadership and governance	Post-conflict and developing states

### Analysis and synthesis of results

Following the gender analysis framework, we assessed the content, including sex-segregated data and the utilization of the gender lens by the identified studies, published in peer-reviewed journals. We present the proportion of males and females in each study in Table 2. Only one study [7] and a review paper [40] utilized the gender analysis framework. The following section describes the sources identified for 11 countries, which have reported sex-segregated findings and factors affecting women’s participation in the health workforce.

#### Afghanistan

Afghanistan made substantial progress regarding women’s representation in the health workforce in the post-conflict period, which has increased to almost 47% in 2016 from 21% in 2002 [18, 41]. Two empirical sources are part of this review, including a study that described the challenges faced by nurse administrators in Kabul, including the lack of female nurses in leadership positions and the health workforce [16]. Even with a substantial increase in the number of females in the health workforce, the proportion of female nurses (18%) and physicians (22%) remains low [18]. A literature review reports that targeted recruitment and deployment of the community midwifery and nursing programs seemed successful in closing the gender and geographic imbalances; still, several barriers, including insecurity, cultural and financial issue inhibit equitable distribution of, and access to, health workers in rural regions. Issues arising from differential remuneration of local health care works in programs supported by international donors were also highlighted [17, 38].

#### Sierra Leone

Sierra Leone experienced 10 years of conflict from 1991 to 2002. Since then, several reforms and policies, although drafted, including a national health policy (2002), HRH development plan (2004–2008), and policy (2006), have been challenging to implement until the introduction of the free health care initiative in 2009 because they were driven by external agencies and lacked national ownership [42]. In 2016, female representation in the entire health workforce reached 62% [39]; females in nursing contributed to 90% [19], compared to 30% female physicians [39]. The six research studies included Sierra Leone, comprise two review papers, including a systematic review which followed a framework synthesis approach analyzing health system’s interventions for gender equity in the HRH in FCASs, reported that no strategy developed promoting gender equity in the health workforce [11, 43]. A mixed-methods study to understand gender influences on the health workforce reported predominance of females in nursing, but decision-making positions in nursing are also occupied by males [7].

Of the three qualitative studies, one captured experience and resilience by staff reported several effects of conflict which were complicated by epidemics of the Ebola virus that created difficulties for women to continue their jobs because of stigma and pressure from family members [44]. Another study explained the gendered norms of “caring,” a ‘feminine character’ that served as both a trigger and an expectation for females to become health professionals and stay in service, particularly nurses [45]. In addition, women were discouraged from taking their families when relocated [7] caused personal insecurity and emotional strain among women [11, 46].

#### Timor-Leste

The initial post-conflict period in Timor-Leste (1999–2002) witnessed a shortage of health workforce due to staff fleeing. An analysis of the policy-making processes for HRH recruitment over 20 years revealed that the involvement of Cuban Medical Brigades (CMB) was helpful in the recruitment and deployment of the HRH, considering gender and geographic location, serve as a game changing period (2003–2005). The CBM played an instrumental role in creating a recruitment system, particularly for physicians, and provided pre-service training to physicians from Timor-Leste at the Latin Medical School in Cuba, resulted in 1.01 male to female ratio, but was higher for males (2.6) at specialist level [47]. Another study for understanding the labor market dynamic among physicians, nurses, and midwives did not identify gender as a limiting factor in training, remuneration, and supervision. It was interesting to note that the majority of participants (60%) in the study were female, and males (70%) dominated nursing workforce [48], which is similar to 62% of nurses being male at the national level [19]. Although at the national level plans emphasized gender mainstreaming, no strategies had been developed to translate those plans and promote gender equity in the HRH. This is primarily due to insufficient consultation of national stakeholders by consultants and donors [11] and the lack of intersectoral collaboration [47].

#### Mozambique

Mozambique was included in two studies: the first investigated gender differences in accessing non-pecuniary benefits, and women had significantly fewer opportunities for professional development representing 40% female physicians and 56% female nurses and midwives [49]. The second examined the percentage of female physicians in three capital cities, including, Bissau (Guinea-Bissau) and Maputo (Mozambique); women physicians, contributing to 46.2% across the cities, with 51% in Maputo. Among the three countries, Mozambique had a significant salary disparity with male physicians earning significantly more than their female colleagues [50]. This earning disparity could be accounted for by physicians (mostly males) moving into private practice, a trend which places additional pressure on public sector nurses and midwives, mostly females [11]. A literature review on health system reconstruction reported promoting gender equity within the ministry of health by appointing gender focal personnel to integrate gender in health policies. However, the limited capacity of the focal personnel and limited scope of gendered interventions to maternal conditions could not respond to the health needs of women across the lifespan. Regarding prioritizing gender in HRH, while Mozambique’s university medical school was committed to training more female physicians, no evidence was found for gender equity in the overall HRH strategy [11].

#### Zimbabwe

Zimbabwe’s civil war ended in 1980, but its history of economic recession and political and social crisis between 1997 and 2009 led to poverty and poor standards and systems due to the hyper-inflation leading to demonetization and adoption of multiple currencies [44]. Zimbabwe was part of a study on gender influence on the health workforce [7], which found a strong gendered pattern with nursing and midwifery dominated by females compared to the males, who dominated medical profession. This pattern was justified by the informants who asserted gender stereotypes concerning women’s suitability to front-line care, versus poor performance of men in these roles. For instance, Witter et al. [7] cite a key informant in Zimbabwe, who said that “male nurses were difficult and inefficient, while female nurses are efficient and a marvel to work with” (p.v56). A study on gender differences in access to non-pecuniary benefits reported that access to advance professional training was significantly lower for women [49]. Of three qualitative studies, one captured staff insights on their resilience [44], a second scrutinized implementation of deployment strategies [51], and a third examined staff motivation to join the health professions and retention [45].

Another study examined the realities, challenges, and opportunities for women’s leadership in the field of global health [1]. These studies report that females dominate in the nursing workforce, while males dominate in the medical workforce [7, 49]; males also dominated leadership positions [1, 7, 51]. Motivating factors for females included “passion” and “calling” [7, 45] as compared to males who regarded finances to support family and education as motivation to become and remain as health professionals [7]. Although the pragmatic and flexible approaches of secondment and transfer of the HRH to rural districts was helpful for equitable distribution of staff [51], security [7] and gendered socio-cultural norms and responsibilities affected women's access to professional development and continuing their job in rural settings [1, 7]. For instance, employment in rural locations favored men as they can travel independently, and women had to resign from their jobs to accompany their husbands [7]. A synthesis paper of nine studies on intersectional approaches in research in LMIC included a study from Zimbabwe, reported the intersection of marital status and gender affected women disproportionately for their professional development [40].

#### Chad

Gupta and Alfano [49] investigated gender differences in accessing non-pecuniary benefits among the six LMIC countries, and Chad had the lowest number of female participants for medicine (11%) and nursing and midwifery (24%). Although it was found that did women receive transportation allowances and health insurance in the six LMIC, fewer women received these benefits than men in Chad. Furthermore, a small qualitative study by Jaeger et al. [52] found that female nurses were particularly concerned about training opportunities, basic commodities, and unstable phone networks, the latter of which created strained family relations and security challenges in remote regions.

#### Côte d’Ivoire

Côte d'Ivoire was also included in the study by Gupta and Alfano which investigated gender differences in access to non-pecuniary benefits in six LMIC. They found that more females have access to meals and transportation allowances, paid vacation, and health insurance as compared to their male counterparts. On the other hand, fewer women had access to in-service training and housing allowance. Female participants in the study comprised 21% of physicians and 29% of nurses and midwives [49].

#### Iraq

Iraq has experienced political unrest for more than three decades. Two studies described health system reconstruction priorities for nurses [53] and physicians [54]. The studies found that the perspective on prioritization of health system's reconstruction needs differed with regard to gender and geographic location, and women did not prioritize salary or update the curriculum for medical education [54]. The choice of a specialty area for the practice among nurses was significant, and female nurses significantly predominated in obstetrics/ gynecology (100%) and pediatrics (85.5%) compared to males, who dominated in all other departments [53]. Insecurity was found to deleteriously affect women’s employment in the health workforce [53].

#### Lebanon

Lebanon has experienced multiple wars and civil unrest for three decades, which has destroyed the public health system and fostered a robust private system [13]. A study examined the influence of gender in selecting females in a residency program found that 90% of 22 program directors (82% were male) perceived that marriage and motherhood affect residents’ productivity [55]. Another study on primary healthcare and community stakeholders' perspectives on HRH found a shortage of community health nurses and female gynecologists in rural regions due to insecurity leading to gender imbalance in the health workforce, preventing women from seeking health care [13]. A cross-sectional study of migrated nurses found a significant association between gender and the reasons behind the migration of nurses. Male nurses rated salary (93 vs. 58%), better work opportunities (71 vs. 53%), and career advancement (69 vs. 56%) higher than females. For female nurses, moving with the family was a significant reason (30 vs. 3.4%). The authors rationalized the difference in the ratings due to the Arab cultural norm, which expects males to be the primary breadwinner [56].

#### Guinea-Bissau

Guinea-Bissau's health system is experiencing a severe financial crisis and is dependent on donor funding [50]. A descriptive study by Russo et al. [50] analyzed the percentage of female physicians to understand the implications of feminization of the medical workforce in three countries, including Guinea-Bissau. Despite a progressive feminization of the medical workforce, women only contributed to 28% of the medical workforce, which was the lowest among the three countries [50]. Although the dual practice (simultaneously public and private) was a preferred modality for both female and male physicians, overall, women worked fewer hours in their professional practice and were less likely to hold a specialty. Those who did have a practice speciality were concentrated in pediatrics, general practice, and gynecology and were absent from surgery, orthopedics, stomatology, and otorhinolaryngology [50].

#### Mali

Mali has experienced multi-dimensional ongoing threats since its independence, which have been complicated by armed conflict and political unrest since 2012 [57]. A qualitative study on factors related to poor retention of health workers in rural districts highlighted challenges to the female workforce related to social norms, personal security, and poor living conditions. Women faced strong pressure from their husbands and families to leave rural regions to re-join their husbands, bringing challenges to women’s career progress. Based on the findings, the authors suggested adopting strategies considering the social norms, such as relocating spouses of civil servants to enhance retention of married staff in rural regions [12].

## Discussion

Of the 36 FCASs, we found literature for only 11 countries. More than 80% of identified articles were published after 2014, and in these studies, more than 50% of participants (nurses, physicians and midwives) were female compared to earlier studies, which was less than 50%. The increased representation of females in these studies is indicative of feminization of the health workforce in FCASs and that women are gaining more attention to meet the targets of global goals for UHC and SDGs by 2030 [2, 37, 50]. However, women’s employment remains concentrated in lower-paid occupations such as nursing and midwifery [7, 44, 45, 49, 51, 53, 56] as compared to men who dominate in medicine in most FCASs. Women are less likely to hold a specialty, although they are well represented in pediatrics, general practice, and gynecology. More research is called for to track the impact of the feminization of medicine [50, 55]. Furthermore, decision-making and leadership positions are overwhelmingly occupied by men [1, 7, 16, 47, 51, 55]. These findings are consistent with the gender and equity analysis of the health and social workforce by WHO’s gender equity hub which found widespread adherence to traditional gender norms in HRH [58]. In addition, studies found the lack of women in decision-making positions limited women's voice at policy levels with resultant gender-blind HRH policies [1, 11, 43]. These findings emphasize the need for HRH policies and strategies that take into account gendered socio-cultural norms and gender equity [8, 12, 43].

While nursing remains dominated by women, an interesting pattern of increased representation of men in nursing was observed in several FCASs [16, 19, 48]. While there may be benefits to the professional status of nursing from increased male participation [16, 53], there are concerns that in highly patriarchal societies, male nurses will gain preferential access to senior and leadership positions which in turn could lead to increased migration of female nurses to other countries [56] and professions [16] in FCASs. Therefore, further investigations are suggested to explore the career motivations of male nurses in these FCASs and to determine the maintenance/reproduction of patriarchy in these contexts.

Cultural norms affected the recruitment and mobility of women in the health workforce [7, 17]. Women bear the burden of inequitable gendered expectations and stereotypes from their supervisors (most cases men), such as the beliefs that women are suited to caring behavior while men are equipped to live and travel alone in challenging environments [1, 7]. Studies found that women were required to resign from their jobs and accompany their husband upon relocation [7], while women were discouraged from being accompanied by their family when they assumed a new position [44, 46] creating strained family relations [12, 52]. Similarly, women in the health workforce were penalized for their reproductive role, as male managers perceived that maternal responsibilities negatively affect women's productivity [1, 55]. Contrary to the views of managers, women were found to perceive education and career as a priority; believe they could simultaneously manage family and education [55] and were willing to take on challenges and leadership positions [1]. These findings recommend the creation of gender equitable opportunities for career development in the health workforce [58].

Some sources highlighted that the post-conflict period serves as a window of opportunity to break down gender biases and stereotypes due to renewed political will and to leverage support from international aid and development agencies [7, 11, 36]. Other research highlighted influences by non-state actors, such as NGOs and private sectors attracting and retaining the workers, including females, that negatively affect staffing in governmental institutions [8, 17] and create sustainability issues. Percival et al. [11] highlighted insufficient consultation of national stakeholders by consultants and donors for policy reforms. Despite the availability of resources, donors and consultants were found to narrowly focused on programs and interventions related solely to women's reproductive and sexual health, rather than taking a comprehensive approach to gender mainstreaming in the health system's restructuring, including HRH. These findings suggest consulting relevant national stakeholders, including women, during HRH planning within the FCASs to result in a gender-sensitive health system.

### Limitations

For feasibility reasons we excluded sources for countries not included in the WB's harmonized list of 2018 and 2019. Furthermore, the search strategy included English language publications only and studies that provided sex-segregated data. And while this review's findings cannot be generalized to the entire FCASs, learning from these settings could be applied to similar contexts. Furthermore, the identified studies varied in methods and content, not merely gendered aspects of HRH. Due to scarcity of sources, we included studies highlighting the processes of either recruitment, deployment, retention, or leadership in the health workforce in the post-conflict period during health system reconstruction in general in FCASs. National-level sex-segregated data for the entire health workforce was seldom presented. This deficiency was noted by multiple authors who called for additional context-specific research using sex-disaggregated data [1, 8, 49, 50, 55] in order to understand the dynamics of the health system with regard to gender and professional categories [8, 17, 49] and geography [17, 46].

## Conclusion

The growing literature in the FCASs revealed that women are over-represented in the HRH in the lower valued professions, mostly in nursing and midwifery. In some instances women have overcome the obstacles to become and remained in the health workforce, in general women struggle to function to their full capacity due to constraints related to professional hierarchies, gendered socio-cultural norms in the family and healthcare system, and security conditions in FCASs. Despite the post-conflict period providing a window of opportunity to break down gender biases and stereotypes in the context of resource availability, most FCASs fail to address gender equity in HRH planning because of the lack of intersectoral collaboration, insufficient consultation with national stakeholders by consultants and donors, and influences by non-state actors. FCASs need a comprehensive approach to gender mainstreaming in restructuring the healthcare system, particularly HRH, rather than a narrow focus on women's reproductive health. Without this, equal participation of women in the health workforce and progress towards achieving the SDG on gender equality by 2030 remains a parable.

## Supplementary Information

**Additional file 1. **Sex-segregated data from global health observatory, WHO for the identified FCASs in different years.

**Additional file 2. **World Bank’s (WB) harmonized lists of FCASs for 2018 and 2019.

**Additional file 3. **PRISMA-ScR checklist.

**Additional file 4. **The search strategy of the review and number of resulted sources from MEDLINE and CINHAL databases.

## Data Availability

The complete reference list of sources analyzed for this review is included in this article. However, the full-text articles used as data in this review are available from the corresponding author on reasonable request.

## Abbreviations

FCASs: Fragile and conflict-affected statutes/countries
HRH: Human resources for health
LMIC: Low- and middle-income countries
SDGs: Sustainable Development Goals
UHC: Universal health coverage
WB: World Bank
WHO: World Health Organization
NGOs: Non-governmental organizations
MMR: Maternal mortality rate
RinGs: Research in Gender and Ethics
GHO: Global Health Observatory
