# Supplementary material for: Participation of women in the health workforce in the fragile and conflict-affected countries: a scoping review
Source: Hum Resour Health. 2021 Aug 4;19:94. doi: 10.1186/s12960-021-00635-7 (PMC8336014; doi:10.1186/s12960-021-00635-7)
Supplement: Supplementary file 1 — Additional file 1. Sex-segregated data from global health observatory, WHO for the identified FCASs in different years. [file 12960_2021_635_MOESM1_ESM.docx]

**Table 1: Data from Global Health Observatory (GHO): Sex-distribution of Nursing workforce for different years**

| S.No | Countries | Year | Sex Distribution (%age) | |
| --- | --- | --- | --- | --- |
|  |  |  | **F** | **M** |
|  | Afghanistan | 2016^#^ | 18 | 82 |
|  | Chad | 2019 | 10 | 90 |
|  | Cote d’Ivior | 2018 | 46 | 54 |
|  |  | 2013 | 40 | 60 |
|  | Guinea-Bissau | 2018 | 68 | 32 |
|  |  | 2013 | 61 | 39 |
|  | Iraq | 2018 | 52 | 48 |
|  |  | 2013 | 37 | 63 |
|  | Lebanon | 2018 | 79 | 21 |
|  |  | 2013 | 81 | 19 |
|  | Mali | 2018 | 43 | 57 |
|  |  | 2009 | 60 | 40 |
|  | Mozambique | 2019 | 50 | 50 |
|  |  | 2013 | 68 | 32 |
|  | Sierra Leone | 2018 | 90 | 10 |
|  |  | 2013 | 90 | 10 |
|  | Timor-Leste | 2019 | 38 | 62 |
|  |  | 2017 | 37 | 63 |
|  | Zimbabwe | 2018 | 76 | 24 |
|  |  | 2012 | 79 | 21 |

**Table 2: Data from Global Health Observatory (GHO): Sex-distribution of physician workforce for different years**

| S.No | Countries | Year | Sex/Gender Distribution | |
| --- | --- | --- | --- | --- |
|  |  |  | **F** | **M** |
|  | Afghanistan | 2016^#^ | 22 | 78 |
|  | Cote d’Ivior | 2014 | 19 | 81 |
|  | Lebanon | 2014 | 25 | 75 |
|  | Mali | 2018 | 11 | 89 |
|  |  | 2009 | 24 | 76 |
|  | Mozambique | 2019 | 48 | 52 |
|  | Timor-Leste | 2019 | 48 | 52 |
|  |  | 2013 | 61 | 39 |
|  | Zimbabwe | 2018 | 28 | 72 |
|  |  | 2012 | 32 | 68 |

*GHO (WHO, 2021) | By category | Sex distribution of health workers*. (n.d.). WHO; World Health Organization. Retrieved June 23, 2021, from https://apps.who.int/gho/data/node.main.HWFGRP_BYSEX?lang=en

^#^ *National Health Strategy 2016‒2020*, Ministry of Public Health (MoPH), Afghanistan (MoPH, 2016)
