# Supplementary material for: Participation of women in the health workforce in the fragile and conflict-affected countries: a scoping review
Source: Hum Resour Health. 2021 Aug 4;19:94. doi: 10.1186/s12960-021-00635-7 (PMC8336014; doi:10.1186/s12960-021-00635-7)
Supplement: Supplementary file 2 — Additional file 2. World Bank’s (WB) harmonized lists of FCASs for 2018 and 2019. [file 12960_2021_635_MOESM2_ESM.pdf]

Harmonized List of Fragile Situations FY 18 a/

Country	WBG CPIA	AfDB or ADB CPIA	Harmonized Average	Peacekeeping Missions b/	Political & Peacebuilding Missions c/
**IDA Eligible**					
Afghanistan	2.717	2.783	2.75		P
Burundi	2.975	3.113	3.04		P
Central African Republic	2.417	2.476	2.45		P
Chad	2.742	3.230	2.99		
Comoros	2.858	2.124	2.49		
Congo, Dem. Rep.	2.917	3.246	3.08	PK	
Congo, Rep	2.850	3.281	3.07		
Côte d'Ivoire	3.350	3.713	3.53	PK	
Djibouti	2.967	3.298	3.13		
Eritrea	1.850	2.132	1.99		
Gambia, The	2.925	2.943	2.93	PK	
Guinea-Bissau	2.458	2.628	2.54		P
Haiti	2.875		2.88	PK	
Kiribati	2.950	2.942	2.95		
Kosovo	3.567		3.57	PK	
Liberia	3.100	3.357	3.23	PK	
Mali	3.358	3.741	3.55	PK	
Marshall Islands	2.600	2.875	2.74		
Micronesia, Fed. Sts	2.750	2.883	2.82		
Mozambique	3.233	3.165	3.20		
Myanmar	3.075	3.308	3.19		
Papua New Guinea	3.000	2.858	2.93		
Sierra Leone	3.242	3.312	3.28		P
Solomon Islands	2.975	3.225	3.10		
Somalia	1.750	1.195	1.47		P
South Sudan	1.575	1.858	1.72	PK	
Sudan	2.467	2.560	2.51	PK	
Syria					
Togo	3.025	3.188	3.11		
Tuvalu	2.858	3.017	2.94		
Yemen, Rep.	2.383		2.38		
**Territories**					
West Bank and Gaza					P
**Blend**					
Zimbabwe	2.717	2.660	2.69		
**IBRD Only**					
Iraq					P
Lebanon					P
Libya					P

a/ "Fragile Situations" have: either a) a harmonized average CPIA country rating of 3.2 or less, or b) the presence of a UN and/or regional peace-

b/ Specifically defined as the presence of a UN and/or regional (eg: AU, EU, OAS, NATO) peace-keeping operation in this country in the last three years, with the exclusion of border monitoring operations [sources: UN DPKO, AU, EC, websites] For additional information regarding this list, please read the FCS Information Note and FAQ found on our website: www.worldbank.org/fragilityandconflict

c/ Specifically defined as the presence of a UN and/or regional (eg: AU, EU, OAS) peace-building and political mission in this country in the last three years [sources: UN DPKO, AU, EU websites]

Harmonized List of Fragile Situations FY 19 a/						
Country	WBG CPIA	ADB CPIA	AFDB CPIA	Harmonized Average	Peacekeeping Missions b/	Political & Peacebuilding Missions c/
**IDA Eligible**						
Afghanistan	2.68	2.78		2.73		P
Burundi	2.91		3.11	3.01		
Central African Republic	2.48		2.48	2.48		P
Chad	2.66		3.23	2.94		
Comoros	2.81		2.12	2.46		
Congo, Dem. Rep.	2.83		3.25	3.04	PK	
Congo, Rep	2.70		3.28	2.99		
Côte dIvoire	3.38		3.71	3.54	PK	
Djibouti	2.97		3.30	3.13		
Eritrea	1.85		2.13	1.99		
Gambia, The	2.97		2.94	2.95	PK	
Guinea-Bissau	2.45		2.63	2.54		P
Haiti	2.88			2.88	PK	
Kiribati	2.99	2.94		2.97		
Kosovo	3.57			3.57	PK	
Liberia	3.13		3.36	3.24	PK	
Mali	3.40		3.74	3.57	PK	
Marshall Islands	2.60	2.88		2.74		
Micronesia, Fed. Sts	2.75	2.88		2.82		
Mozambique	3.17		3.16	3.16		
Myanmar	3.00	3.31		3.15		
Solomon Islands	2.93	3.23		3.08		
Somalia						P
South Sudan	1.53		1.86	1.69	PK	
Sudan	2.38		2.56	2.47	PK	
Syria						
Togo	3.13		3.19	3.16		
Tuvalu	2.90	3.02		2.96		
Yemen, Rep.	2.11			2.11		
**Territories**						
West Bank and Gaza						P
**Blend**						
Papua New Guinea	2.96	2.86		2.91		
Timor-Leste	2.85	3.46		3.15		
Zimbabwe	2.78		2.66	2.72		
**IBRD Only**						
Iraq						P
Lebanon						P
Libya						P

a/ "Fragile Situations" have: either a) a harmonized average CPIA country rating of 3.2 or less, or b) the presence of a UN and/or regional peace-keeping or peace-building mission during the past three years. This list includes only IDA eligible countries and non-member or inactive territories/countries without CPIA data. IBRD countries with CPIA ratings below 3.200 do not qualify on this list due to non disclosure of CPIA ratings; IBRD countries that are included here qualify only by the presence of a peacekeeping, political or peace-builing mission - and their CPIA ratings are thus not quoted here

b/ Specifically defined as the presence of a UN and/or regional (eg: AU, EU, OAS, NATO) peace-keeping operation in this country in the last three years, with the exclusion of border monitoring operations [sources: UN DPKO, AU, EC, websites] For additional information regarding this list, please read the FCS Information Note and FAQ found on our website: www.worldbank.org/fragilityandconflict

c/ Specifically defined as the presence of a UN and/or regional (eg: AU, EU, OAS) peace-building and political mission in this country in the last three years [sources: UN DPKO, AU, EU websites]
