# Supplementary material for: Participation of women in the health workforce in the fragile and conflict-affected countries: a scoping review
Source: Hum Resour Health. 2021 Aug 4;19:94. doi: 10.1186/s12960-021-00635-7 (PMC8336014; doi:10.1186/s12960-021-00635-7)
Supplement: Supplementary file 4 — Additional file 4. The search strategy of the review and number of resulted sources from MEDLINE and CINHAL databases. [file 12960_2021_635_MOESM4_ESM.docx]

**Search Results: MEDLINE Database**

| **S.No** | **Searches** | **Results** |
| --- | --- | --- |
| 1 | dentists, women/ or physicians, women/ or women, working/ | 11661 |
| 2 | Female/ | 8216211 |
| 3 | female.tw,kf. | 640309 |
| 4 | Wom*.tw,kf. | 1085598 |
| 5 | Woman.tw,kf. | 197724 |
| 6 | ((wom* or Female) adj4 (participat* or decision*)).tw,kf. | 4206 |
| 7 | (wom?n* adj3 empowerment).tw,kf. | 1032 |
| 8 | (Gender adj4 (equality or equity or analys*OR discriminatio*)).tw,kf. | 1672 |
| 9 | 1 or 2 or 3 or 4 or 5 or 6 or 7 or 8 | 8478803 |
| 10 | health personnel/ or allied health personnel/ or dental assistants/ or dental hygienists/or emergency medical technicians/ or anesthetists/ or anesthesiologists/ or nurse anesthetists/ or exp health facility administrators/ or exp nurses/ or exp nursing staff/ or occupational therapists/ or personnel, hospital/ or exp physicians/ | 382929 |
| 11 | allied health personnel/ or anesthetists/ or faculty, medical/ or faculty, nursing/ or health educators/ or health facility administrators/ or medical staff/ or nurses/ or nursing staff/ or personnel, hospital/ or physical therapists/ or exp physicians/ | 267724 |
| 12 | Health workforce.tw,kf. | 2682 |
| 13 | Health Human resource.tw,kf. | 126 |
| 14 | Human resources for health.tw,kf. | 810 |
| 15 | Female nurs*.tw,kf. | 1171 |
| 16 | Female Physician*.tw,kf. | 1296 |
| 17 | Midwi*.tw,kf. | 23440 |
| 18 | ((health or Healthcare or Health Care) adj3 (workforce or Personnel or provider* or Nurs* or midwi* or doctor* or decision maker*)).tw,kf. | 115444 |
| 19 | 10 or 11 or 12 or 13 or 14 or 15 or 16 or 17 or 18 | 518065 |
| 20 | "warfare and armed conflicts"/ or armed conflicts/ or afghan campaign 2001-/ or iraq war, 2003-2011/ or russian-japanese war/ or september 11 terrorist attacks/ or psychological warfare/ | 5210 |
| 21 | "Fragile and conflict affected State*".tw,kf. | 15 |
| 22 | Post?conflict countries.tw,kf. | 5 |
| 23 | Health system reconstruction.tw,kf. | 15 |
| 24 | ((conflict or fragil* or Post?conflict) adj4 (Health system* or situation* or State* or Context* or countr*)).mp. [mp=title, abstract, original title, name of substance word, subject heading word, floating sub-heading word, keyword heading word, organism supplementary concept word, protocol supplementary concept word, rare disease supplementary concept word, unique identifier, synonyms] | 2990 |
| 25 | ((health system or community health) adj3 (program or reconstruction)).tw,kf. | 691 |
| 26 | 20 or 21 or 22 or 23 or 24 or 25 | 8820 |
| 27 | 9 and 19 and 26 | 295 |

**Search Result: CINHAL Database**

| **Search Options** | **Actions** | **Number** |
| --- | --- | --- |
| S1 | (MH "Women") OR (MH "Physicians, Women") OR (MH "Women, Working+") | (22,022) |
| S2 | TI ( Wom?n OR Female ) OR AB ( Wom?n OR Female ) | (454,142) |
| S3 | TI ( (wom?n* N3 empowerment OR decision make*OR leader*) ) OR AB ( (wom?n* N3 empowerment OR decision make*OR leader*) ) | (595) |
| S4 | TI ( TI (Gender N4 equality OR equity OR analys*OR discriminatio*) ) OR AB ( TI (Gender N4 equality OR equity OR analys*OR discriminatio*) ) | (2,925) |
| S5 | S1 OR S2 OR S3 OR S4 | (463,027) |
| S6 | (MH "Personnel Recruitment") OR (MH "Personnel Retention") OR (MH "Personnel Turnover") OR (MH "Nursing Manpower+") OR (MH "Physician Incentive Plans") OR (MH "Promotion and Tenure") OR (MH "Salaries and Fringe Benefits") OR (MH "Family and Medical Leave") | (249,975) |
| S7 | TI (Health workforce OR Health Human resource OR Human resources for health OR Female nurse* OR Female Physician* OR Midwi* OR health worker*) OR AB (Health workforce OR Health Human resource OR Human resources for health OR Female nurse* OR Female Physician* OR Midwi* OR health worker* ) | (38,124) |
| S8 | TI ( ((health OR Healthcare OR Health Care) N3 (workforce OR Personnel OR health worker* OR provider* OR Nurs* OR midwi* OR doctor* OR decision maker*)) ) OR AB ( ((health OR Healthcare OR Health Care) N3 (workforce OR Personnel OR health worker* OR provider* OR Nurs* OR midwi* OR doctor* OR decision maker*)) ) | (98,166) |
| S9 | S6 OR S7 OR S8 | (355,356) |
| S10 | TI ( (("Fragile N2 conflict affected States") OR (Post-conflict N2 health system) OR (Health system N2 reconstruction)) ) OR AB ( (("Fragile N2 conflict affected States") OR (Post-conflict N2 health system) OR (Health system N2 reconstruction)) ) | (16) |
| S11 | TI ( ((conflict OR fragil* OR Post?conflict) N4(Health system* OR situation* OR State* OR Context* OR countr*)) ) OR AB ( ((conflict OR fragil* OR Post?conflict) N4(Health system* OR situation* OR State* OR Context* OR countr*)) ) | (1,304) |
| S12 | TI ( ( "Fragile conflict affected States" OR South Sudan OR Somalia OR Yemen OR Syria OR Central African Republic OR Congo Democratic Republic OR Sudan OR Chad OR Afghanistan OR Zimbabwe OR Iraq OR Haiti OR Papua New Guinea OR Guinea Bissau OR Eritrea OR Myanmar OR Cameroon OR Burundi OR Cote divoire OR Djibouti OR Gambia OR Kiribati OR Kosovo OR Liberia OR Mali OR Marshall Island OR Micronesia OR Mozambique OR Solomon Islands OR Togo OR Tuvalu OR Timor-Leste OR Lebanon OR Libya Or West Bank OR  [...](javascript:showHistoryTerm('ctl00_ctl00_FindField_FindField_historyControl_HistoryRepeater_ctl11_ellipsis',true)) | (16,436) |
| S13 | S10 OR S11 OR S12 | (17,604) |
| S14 | S5 AND S9 AND S13 | (393) |
